# Ultrasound-assisted preparation of ‘Ready-to-use’ extracts from Radix Paeoniae Rubra with natural deep eutectic solvents and neuroprotectivity evaluation of the extracts against cerebral ischemic/ reperfusion injury

**DOI:** 10.1016/j.ultsonch.2022.105968

**Published:** 2022-03-02

**Authors:** Yu Zhao, Haofang Wan, Jiehong Yang, Yan Huang, Yu He, Haitong Wan, Chang Li

**Affiliations:** Zhejiang Chinese Medical University, Hangzhou 310057, PR China

**Keywords:** Natural eutectic solvent, Radix Paeoniae Rubra, Paeoniflorin, Galloyl paeoniflorin, Ischemic/ reperfusion injury

## Abstract

Natural deep eutectic solvent (NaDES) is widely applied in the extraction of nutrients from natural resources as a greener alternative for fossil solvent. In the present work, 27 different NaDESs were screened for the extraction of paeoniflorin (PF) and galloyl paeoniflorin (GPF) from Radix Paeoniae Rubra (RPR). After screening and extraction parameter optimization, the extraction yields of PF and GPF reached up to 182.8 mg/g and 77.4 mg/g with the selected NaDES, ChCl-Sor. Furthermore, the antioxidant activity *in vitro* and neuroprotectivity *in vivo* of the ‘ready-to-use’ extracts were evaluated comprehensively. Especially *in vivo*, the cerebral ischemic/ reperfusion injury model was established in rats and the protective effects of the RPR extracts were determined. The results not only proved that NaDES is a valuable green extraction media, but also indicated the safety and potential pharmaceutical application of NaDES based ‘ready-to-use’ extracts from medical plants.

## Highlights

• A ready-to-use NaDES extract of RPR was screened out of 27 NaDESs.
• The extraction yields of ChCl-Sor was higher than conventional solvents.
• Four *in vitro* antioxidant assays of the extracts were performed.
• The extract can be directly used in vivo to attenuate cerebral I/R injury.

## Graphical abstract

**Figure:**
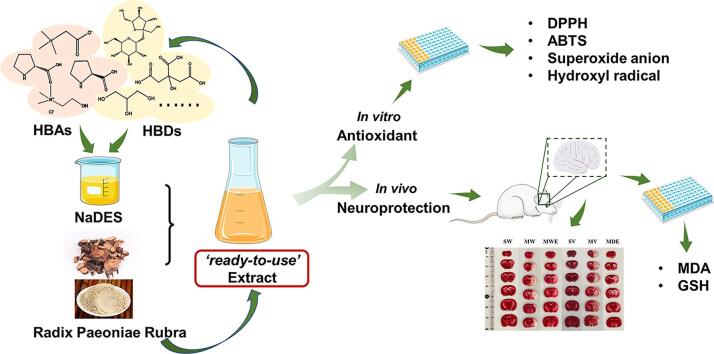

## 1 Introduction

Natural deep eutectic solvent (NaDESs) is a sort of emerging environment-friendly solvents firstly introduced in 2011 [1]. In recent decades, the application of NaDES was greatly expanded, ranging from nano-materials to organic synthesis [2], [3]. In particular, the usage of NaDES in the extraction process of natural products from traditional Chinse medicines (TCM) and other industrial crops is of significant importance [4]. A great variety of natural bioactive compounds, including flavonoids [5], terpenoids [6] and alkaloids [7], [8], *etc.*, have been extracted using NaDES as a sustainable alternative to conventional solvents such as methanol or acetone. The extracts of these herbal products shared some major advantages in common, which were contributed to the unique physicochemical properties of NaDES, including environment-friendly and non-toxic.

**Fig. 1:**
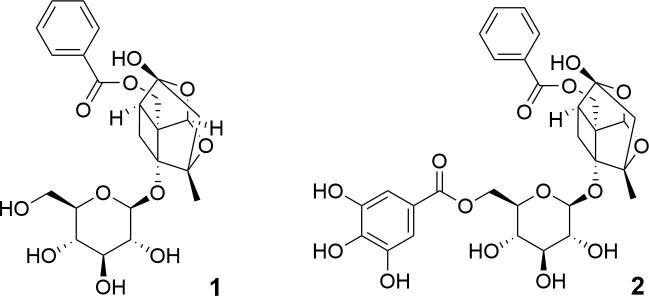
Chemical structures of PF (**1**) and GPF (**2**).

On the other hand, Radix Paeoniae Rubra (RPR, called as *Chishao* in China), the dried root of *Paeonia lactiflora* Pall. and *P. veitchii* Lynch, is a TCM with vast application as herbal medicine or functional food [9], [10]. Clinically, RPR has been applied in treatment of painful conditions, blood stasis treating and menstrual disorders. Pharmacological studies have indicated that RPR possesses anti-thrombotic, hypolipidemic, anti-arteriosclerosis [11] and hepatoprotective effects [12], which made it potential for cardiovascular and cerebrovascular diseases [13], [14]. According to previous studies [15], the mechanisms of these pharmacological effects may be related to the active ingredients in RPR that can inhibit inflammation or antioxidant (Structures shown in Fig. 1). The major constituents in RPR are paeoniflorin (PF) and its derivative, galloyl paeoniflorin (GPF). As previously reported, PF was able to reverse fructose-induced insulin resistance and hepatic steatosis in the treatment of insulin resistance and non-alcoholic fatty liver diseases [16]. In addition, PF also exhibited great potential as an effective therapeutic agent against inflammation [17]. Moreover, GPF was proven as an antioxidant for the treatment of ROS-related disorders including cerebral ischemia [18], [19].

Herein, we prepared 27 different NaDESs with 4 common hydrogen bond acceptors (HBA). Their extraction capacities for PF and derivatives from RPR were investigated. After the best NaDES solvent was screened out, the factors in ultrasonic-assisted extraction (UAE) were further optimized. Four *in vitro* antioxidant evaluation models, namely DPPH, ABTS, hydroxyl radical and superoxide anion scavenging models, were applied to comparatively assess the antioxidative effect of NaDES extracts of RPR. Furthermore, the in vivo neuroprotectivity of the extract against cerebral ischemic/ reperfusion injury was also evaluated in focal middle cerebral artery occlusion (MCAO ) rat models.

## 2 Materials and methods

### 2.1 Chemicals

The dry RPR material was purchased from Zhejiang Tiandao Pharmaceutical Co., Ltd. (Hangzhou, China). PF, GPF, L-ascorbic acid (LAA), nicotinamide adenine dinucleotide (NADH) and nitroblue tetrazolium (NBT) were all purchased from Shanghai Yien Chemical Technology Co., Ltd. (Shanghai, China) with over 98% purity. Rosmarinic acid (RA, 98%) was purchased from Shanghai Macklin Biochemical Co., Ltd. (Shanghai, China). Compounds for NaDES preparation, formic acid (88%), phenazine methosulphate (PMS, 98%) and DPPH were all purchased from Shanghai Aladdin Bio-Chem Technology Co., Ltd. (Shanghai, China). Trizma base (≥99%) was purchased from Sigma-Aldrich (Wuxi) Life Science & Technology Co., Ltd. (Jiangsu, China). HCl was purchased from Yonghua Chemical Co., Ltd (Jiangsu, China). HPLC-grade acetonitrile was purchased from Tedia Co. (Fairfield, USA), and the deionized water used in the study was got from a Milli-Q water purification system (Bedford, USA).

### 2.2 Experimental animals

Adult male SD rats (body weight, 290 ± 10 g) were obtained from the Animal Central of Zhejiang Chinese Medical University, Hangzhou, China (Laboratory animal certificate: SCXK(Zhehe) 2018-0012). All protocols in this study were approved by the Animal Subjects Review Board of Zhejiang Chinese Medical University (No. 20180514-04, 14 May 2018). All animals were cared for according to the National Institutes of Health (NIH) Guide for the Care and Use of Laboratory Animals (National Research Council of the National Academies, 2011). The present research complied with the commonly accepted “3Rs”. The animals were maintained under controlled environmental conditions at 25 ± 1 °C and 60–65 % air humidity under a 12 h light/12 h dark cycle with free access to food and water.

### 2.3 Preparation of NaDESs

All NaDESs were prepared according to our previously reported method [5]. The components of NaDESs were shown in Table S1 in Supporting Information. Briefly, HBA, HBD and water were mixed in corresponding ratios. The mixtures were stirred at 90 °C until a homogeneous liquid was formed. All NaDESs were kept in a desiccator.

### 2.4 Extraction of components with different solvents

Extraction was performed in a clean tube with 1 mL of solvent and 25 mg of RPR powder in an ultrasonic bath (KQ5200DE, Kunshan Ultrasonic Instrument Co.) at 50℃, 50 kHz for 30 min. After centrifugation at 13,300 r/min for 20 min, the suspension was then transferred to another 1.5 mL microtube and diluted with the same volume of methanol–water (1:1) for HPLC analysis. Each extraction was performed by triplicate.

### 2.5 Chromatographic conditions

Quantitative HPLC analysis was performed on an Agilent 1200 system equipped with a G1311A QuatPump, a G1322A degasser, a G1315D diode array detector, and a G1329A ALS with a 20 μL loop. Agilent Extend-C18 (250 mm × 4.6 mm i. d., 5 μm, Agilent) was used to analyze samples. The mobile phase consisted of water with 0.1% formic acid solution (A) - acetonitrile (B) in a linear gradient program as follows: 0–20 min, 10–30% B; 20–25 min, 30–45% B; 25–30 min, 45–95% B; 30–35 min, 95% B at a flow rate of 0.8 mL/min. Chromatograms were recorded at 280 nm. The injection volume was 20 μL.

### 2.6 Extraction parameter optimization

The starting parameters of UAE were set as the same as described in 2.4. In each experiment, one of the following extraction parameters of the UAE process was optimized while the others were kept as the starting condition. Solid/Liquid (RPR powder/ NaDES solution) ratio (mg/mL): 25, 50, 100 and 150; water content in NaDES solution (%): 25, 50, 75 and 100; extraction time (min): 15, 30, 45 and 60; Extraction temperature (℃): 30, 40, 50 and 60.

### 2.7 Antioxidant activities of RPR extracts *in vitro*

#### 2.7.1 DPPH radical scavenging assay

DPPH radical scavenging activity was evaluated by previously described method with modifications [20], [21]. 180 μL of freshly prepared DPPH methanol solution (150 μM) and 20 μL sample or blank solution were added subsequently into a 96-well microplate. The reaction mixture was incubated at 37 °C for 30 min, and the absorbance was measured at 517 nm. L-ascorbic acid (0.6 mM) was used as a positive control. Radical scavenging activity was calculated using the following equation:

$$\mathrm{Scavenging Ratio}\;\left(\%\right)\;=\left(A_{\mathrm{blank}}-\;A_{\mathrm{sample}}\right)/A_{\mathrm{blank}}\;\times 100\%.$$

where A_blank_ is the absorbance of the control reaction mixture without the test samples, and A_sample_ is the absorbance of the reaction mixture containing the test samples. The scavenging ratios were expressed as mean ± SD deviation for three separate experiments.

#### 2.7.2 ABTS radical scavenging activity

ABTS radical scavenging activity was determined with Total Antioxidant Capacity Assay Kit (T-AOC assay Kit, Shanghai Beyotime Biotechnology, China). 20 μL of catalase working solution and 10 μL of sample or blank solution were mixed in a 96-well plate. Then 170 μL of ABTS working solution was added to each well. After incubating for 6 min at room temperature (25 °C), the absorbance was measured at 414 nm. L-ascorbic acid (0.6 mM) was used as positive control. Then the radical scavenging activity was calculated with a similar method as DPPH.

#### 2.7.3 Superoxide anion (O_2_^• −^) scavenging activity

According to the method described by Lim et al. [22], superoxide anion scavenging activity was performed in ice bath. 16 mM Tris-HCl buffer (pH 8.0) was used for the preparation of 300 μM NBT, 468 μM NADH and 60 μM PMS. The superoxide anion was generated by adding respectively 50 μL of NBT, NADH as well as different samples, and started by adding 50 μL of PMS. After 5 min incubation at room temperature, the absorbance was measured at 560 nm. Rosmarinic acid (1 mM) was used as a positive control.

#### 2.7.4 Hydroxyl radical scavenging activity (•OH)

Hydroxyl radical scavenging activity was tested using Hydroxyl free radical assay kits (Nanjing Jiancheng Biotechnology Institute, China) according to the instruction, where Fenton reaction was used to generate hydroxyl radicals [22]. Briefly, Fenton working solution and samples were added in sequence, and the reaction was kept at 37 °C for 1 min then. The Griess developer was immediately added to terminate the reaction. The absorbance was measured at 550 nm after 20 min. L-ascorbic acid (0.9 mM) was used as positive control.

### 2.8 Neuroprotective activities of RPR extracts in rats

#### 2.8.1 Experimental design

A total of 60 adult male SD rats were randomly divided into the six groups (n = 10 per group) as follows: (I) **the sham-water group** was operated with no ischemia and given water as normal; (II) **the sham-vehicle group** was operated with no ischemia and given 75% D-Pro-Sor per day; (III) **the MCAO-water group** was operated with MCAO and given water as normal; (IV) **the MCAO-vehicle group** was operated with MCAO and given 75% D-Pro-Sor per day; (V) **the MCAO-WE group** was administered the RPR water extract (500 mg/kg) and operated with MCAO; (VI) **the MCAO-DE group** was administrated with the RPR D-Pro-Sor extract at a dose of 500 mg/kg and operated with MCAO. The rats in each of the groups were subdivided into two subgroups consisting of 4 and 6 rats respectively. One of the subgroups (4 rats) was used for TTC staining and the other one (6 rats) was used for the evaluation of GSH and MDA levels. The sham group was only subjected to surgical procedures, while other animals were subjected to focal ischemia by MCAO using an intraluminal thread, and after 1 h of MCAO reperfusion was allowed by retracting the thread.

#### 2.8.2 Model establishment

I/R injury was induced by MCAO following Longa’s bolt wire method with slight modifications [23], [24]. In brief, the rats were anesthetized with intraperitoneal injection of 3% pentobarbital sodium (45 mg/kg). Subsequently, the peripheral nerves and tissues are carefully separated to expose the right common carotid artery (CCA), external carotid artery (ECA) and internal carotid artery (ICA). The CCA and ECA were ligated successively, and the ICA was clamped by the arterial clamp. A nylon wire with a rounded tip (diameter of 0.28 mm, Huawei Medical Products Co., Ltd. Hangzhou, China) was carefully inserted into the ICA until the middle cerebral artery (1.8–2.0 cm). During the operation, the room temperature was maintained at 37 ± 1 °C. After 60 min of ischemia, the nylon filament was slowly pulled out for reperfusion. The incision was ligated, sutured and sterilized. The skin incision and blood vessel dissection only were performed in the sham group, and the rest of the operation was the same as the other groups. Rats that showed excessive bleeding and early death in the surgery were excluded.

After reperfusion for 23 h following 1 h of cerebral ischemia, the operated rats were observed and graded. Rats with contralateral hemiparesis and Horner’s syndrome (1 point), contralateral orbiting (2 points), contralateral tumble (3 points), and paralysis without consciousness (4 points) were chosen as stroke rats. The rats with scores in the range of 1–3 points were selected for the test, with 10 rats in each group.

After I/R injury, the rats were administrated intragastrically with 3 mL of the corresponding extracts or vehicles once daily for 6 consecutive days. During the gavage period, food and water were obtained freely. Blood and brain samples collected from rats at 30 min after administration on the 6th day.

#### 2.8.3 Evaluation of infarct volume by TTC staining

TTC staining was applied to estimate the infract volume. Briefly, the collected brains were immediately placed in ice-cold saline for rinsing of the blood. The brain was then dissected into several 2-mm coronal sections and stained by immersion in a 2% 2,3,5, -triphenyltetrazolium chloride (TTC) (Sigma, USA) at 37 °C for 30 min. The staining images were recorded by a digital camera and quantified analyzed by Image J software. The percent of total infract volume was calculated as the white area*2 mm/total area*2 mm × 100% of all brain slides.

#### 2.8.4 Assay of MDA and GPX levels in cerebral cortex

The cortical area corresponding to the ischemic core and penumbra were isolated for biochemical examinations over an ice cube. After weighing, the isolated brain issue was collected in −80℃. The total protein estimations were done using Pierce™ BCA protein assay kits (Thermo Scientific, U.S.). The levels of malondialdehyde (MDA) and glutathione (GSH) were detected using commercial kits (Beyotime Biotechnology, China). The experiment was performed strictly according to the manufacturer’s instructions. Optical absorption was assessed at 530 nm for MDA and 412 nm for GSH using a microplate reader (Bio-Rad Laboratories, Inc., Hercules, CA, USA). Finally, the MDA and GSH concentration was expressed as nmol/mg protein.

#### 2.8.5 Statistical analysis

All data collected in this study were analyzed using GraphPad Prism 8.0 software and SPSS 25.0 software. The data was expressed as the mean ± standard deviation (SD) of independent experiments. The comparison of data between groups were done by one-way analysis of variance (ANOVA) followed by the least significant difference (LSD) test and Tukey post-hoc test for intergroup comparisons. Differences were considered statistically significant at *P* < 0.01.

## 3 Results

### 3.1 Evaluation of NaDESs in extraction of RPR

In present investigation, four typical HBAs were selected, namely choline chloride, betaine, L-proline and D-proline, as well as four kinds of HBDs including sugar, alcohols, amides and carboxylic acids. A total of 27 NaDESs were prepared to evaluated their extraction capacity for PF and GPF.

**Fig. 2:**
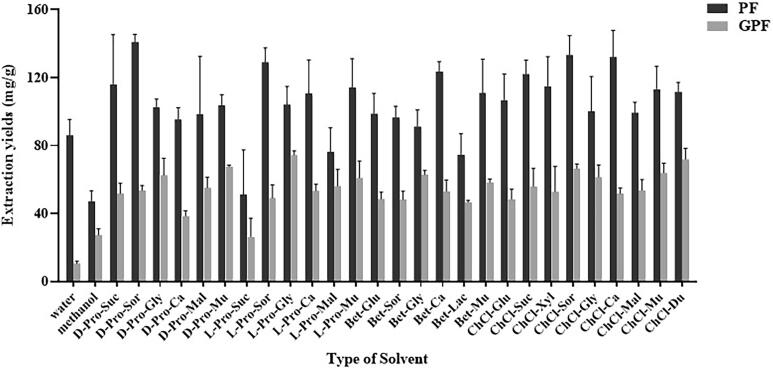
Extraction yields of PF and GPF from RPR using water, methanol and 27 NaDESs. Extraction parameters: 75% NaDES content, S/L ratio 25 mg/mL, extraction temperature 50℃ and extraction time 30 min.

Initially, different NaDESs (75% aqueous solution) were used under UAE, together with water and methanol for comparison. The UAE parameters were S/L ratio 25 mg/mL, extraction temperature 50℃ and extraction time 30 min. The extraction yields were summarized in Fig. 2 (the detailed data was in Table S2 in Supporting Information).

First of all, UAE is applied without preliminary optimization according to the previous examples using NaDES as solvent [25]. Considering the higher viscosity of NaDES comparing with conventional solvents, UAE can provide improved dispersion of extraction solvent and mass transfer efficiency than conventional heating and stirring method. Microwave-assisted-extraction enables faster process, but also causes concern of safety and decomposition of natural compounds. Especially in this case, neither of PF and GPF is thermally stable compound. As the result shows, water exhibited superior extraction capacity for PF (86.0 mg/g), which was 1.8 times that of methanol (47.1 mg/g). In opposition, methanol extracted GPF (27.3 mg/g) much better than that of water (10.6 mg/g). Meanwhile, most of the NaDESs extracted both PF and GPF in high yields. Especially, NaDESs with alcohols or acids as HBDs (*e. g.* Sor, Mal and Mu) exhibited greater potential in extraction of PF, while NaDESs with alcohols or amides (*e. g.* Gly, Sor and Mu) were better for GPF. In the view of HBA, ChCl-based NaDESs (*e. g.* ChCl-Sor, ChCl-Ca and D-Pro-Sor) exerted relatively superior capacity. Therefore, ChCl-alcohol type NaDESs, ChCl-Sor and D-Pro-Sor were selected out for further investigation. Among them, ChCl-Sor is considered to be the optimal solvent based on the total content of PF and GPF (133.3 mg/g and 66.5 mg/g respectively, and 199.8 mg/g in total). In addition, D-Pro-Sor also exhibited good extraction capacity toward PF and GPF, their yields were 140.8 mg/g and 53.6 mg/g, respectively.

### 3.2 Optimization of the extraction factors

**Fig. 3:**
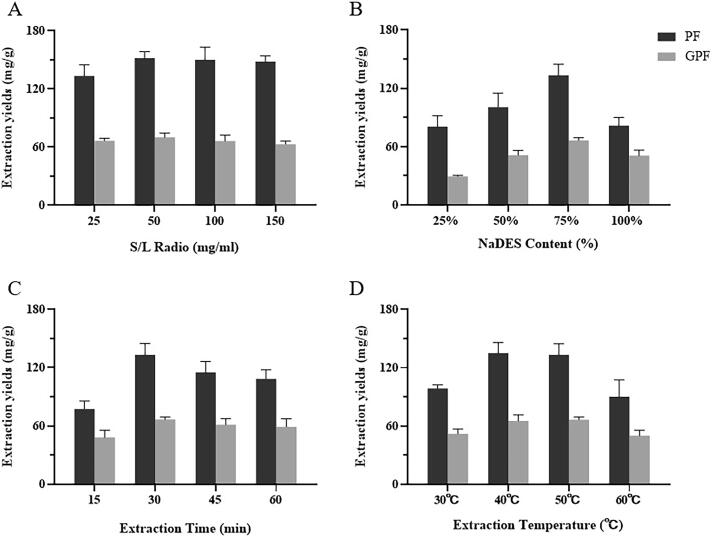
Extraction yields from RPR using ChCl-Sor with different parameters: A) S/L ratio; B) NaDES content; C) Extraction time; D) Extraction temperature.

The UAE parameters were then investigated to optimize the extraction process with ChCl-Sor as extraction solvent. As summarized in Fig. 3, the effects of S/L ratio, NaDES content, extraction temperature and extraction time were presented (the detailed data was in Table S3 in Supporting Information).

The impact of S/L ratio was investigated at 25, 50, 100 and 150 mg/mL while the remaining parameters were kept constant (Fig. 3A). When the S/L ratio reaches 50 mg/mL, the two target components have the highest extraction yield. However, increased S/L ratios did not lead to improved extraction yields. The NaDES content was observed as an important factor to the extraction efficiency, in which 75% NaDES exhibited significant advantages comparing with 25%, 50% and 100% NaDES (Fig. 3C). At the same time, it is shown that increased extraction time and extraction temperature can improve the extraction yield (Fig. 3B & D) until it reaches a certain level. 30 min and 40 ℃ were thus selected as optimal factors, respectively.

To sum up, the optimal extraction factors are chosen as S/L ratio at 50 mg/g, extraction temperature at 40℃, extraction time at 30 min, and NaDES content at 75%. Under this condition, PF and GPF reach their best extraction yields (182.8 mg/g & 77.4 mg/g).

### 3.3 In vitro antioxidant activities of NaDES extracts

#### 3.3.1 DPPH radical scavenging activity

**Fig. 4:**
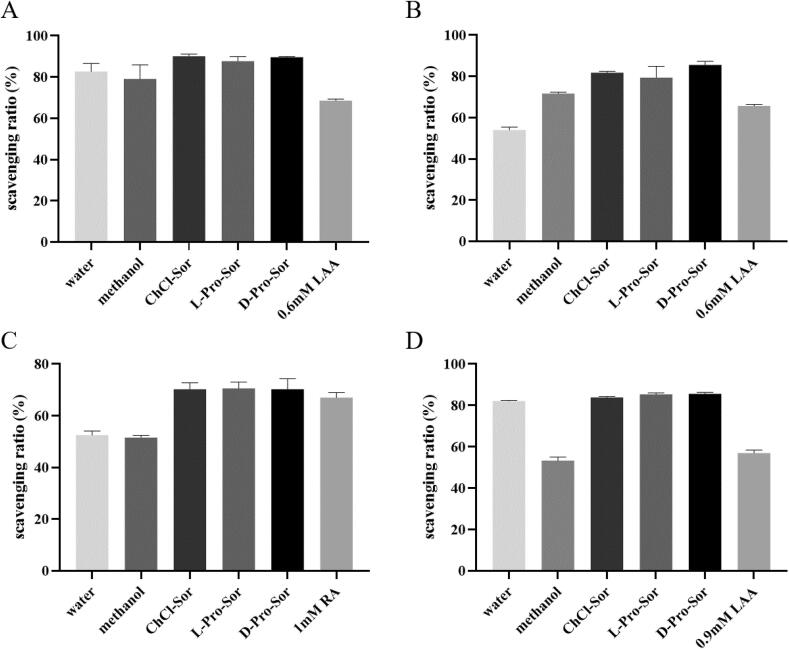
The antioxidant activities of RPR extracts against different radicals. A) DPPH; B) ABTS^+·^; C) superoxide anions and D) hydroxyl radicals.

As presented in Fig. 4, three NaDES extracts were selected to evaluate the *in vitro* antioxidant activities comparing with water and methanol extracts. It could be found that all the selected NaDES extracts of RPR showed strong scavenging capacities against DPPH radical (Fig. 4A). The DPPH scavenging ratios of the NaDES extracts ranged from 87.6% to 90.1% at the concentration of 10 mg RPR/mL, which is slightly better than that of aqueous extracts (82.6%) and much higher than that of methanol extract (79.1%). Additionally, 0.6 mM of LAA as the positive control can scavenge 68.5% DPPH radical in our experiment.

#### 3.3.2 ABTS radical scavenging activity

Analogously, ABTS radical scavenging activity is also considered briefly reflecting the ROS inhibitory effect of antioxidants. As shown in Fig. 4B, the inhibitory effect of NaDES extracts (ranging from 79.4% to 85.5%) on ABTS^+·^ was significantly better than that of water extract (54.0%), as well as methanol extract (71.7%) at the concentration of 5 mg RPR/mL. At the same time, the positive control LAA (0.6 mM) scavenged 65.6% ABTS^+·^.

#### 3.3.3 Superoxide anion (O_2_^•−^) scavenging activity

Superoxide anion is an important type of ROS in lots of biological processes. Comparing with DPPH and ABTS radicals, the assays against O_2_^•−^ could reflect the ‘real’ antioxidant capacity of corresponding substance more directly. All the results were shown in Fig. 4C. It is found that at the concentration of 5 mg RPR/mL, the three NaDES extracts of RPR exhibited almost the same effects toward O_2_^•−^ (∼70%). These results were significantly better than that of the water extract (52.5%) and methanol extract (51.5%). In addition, 1 mM of RA, which was selected as the positive control, quenched 76.4% superoxide anions in the same assay.

#### 3.3.4 Hydroxyl radical scavenging activity (·OH)

Hydroxyl radical, which is another important ‘real’ ROS, was also testified as a target herein. In this work, Fenton reaction was employed to generate ·OH radicals. As presented in Fig. 4D, the methanol extract exhibited the lowest hydroxyl scavenging capacity at the concentration of 5 mg RPR/mL (53.3%). Meanwhile, the three NaDES extracts could scavenge ·OH radicals with the ratios ranging from 83.8% to 85.6%. These results are slightly higher than that of RPR aqueous extract (82.1%).

### 3.4 *In vivo* neuroprotectivities of RPR NaDES extracts

#### 3.4.1 Alleviation effects of cerebral I/R injuries by RPR NaDES extracts

**Fig. 5:**
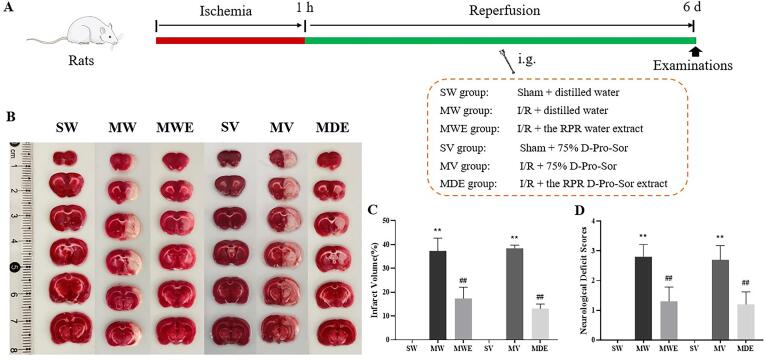
Effects of the RPR extracts on neurological scores and cerebral infarct size in I/R rats. After drug and vehicles treated for 6 d, all rats were graded according to Longa scale, and their brain tissues of four of the rats were transected for TTC staining. (A) Experimental scheme and group design. (B) Representative images of TTC staining in different groups. In brain tissues stained with TTC, red represents normal tissue whereas the infarcted area was white. (C) Quantification of infarct volume per group (n = 4). (D) Quantification of neurological deficit scores per group (n = 10). Values are expressed as mean ± SD. ^**^p < 0.01 as compared with the Normal group. ^##^*p* < 0.01 as compared with the Model group.

As a green alternative to fossil solvents and conventional ionic liquids, one of NaDESs’ advantages is that NaDES extracts are ‘ready-to-use’ extracts due to their low toxicity [26]. As most NaDESs were prepared from food-grade compounds, there have been increasing evidences that it was not necessary to remove the NaDES from the extracts before subjected to experimental animals. Nevertheless, some recently published researches revealed that the uptake of NaDESs together could result in some biological benefits [27], such as improvement of bioavailability and safety [28], protectivity against gastric ulcers [29], and upregulation of some important proteins [30]. Herein, we selected D-Pro-Sor extracts of RPR to evaluated its pharmacological effects against cerebral I/R injury in rats. The TTC staining of brain slices and the scores of neurological deficit after 6 days administration in Fig. 5 showed clearly that treatment with only D-Pro-Sor (group SV & MV) have no influence on the rats both in sham and MCAO group compared with group SW and group MW. However, treatment with RPR aqueous extract (group MWE) and D-Pro-Sor extract (group MDE) for six days significantly alleviate the injury with markedly attenuation of cerebral I/R induced infarct zone and significantly restored of the nerve function.

#### 3.4.2 Effects of D-Pro-Sor extract on endogenous redox markers

**Fig. 6:**
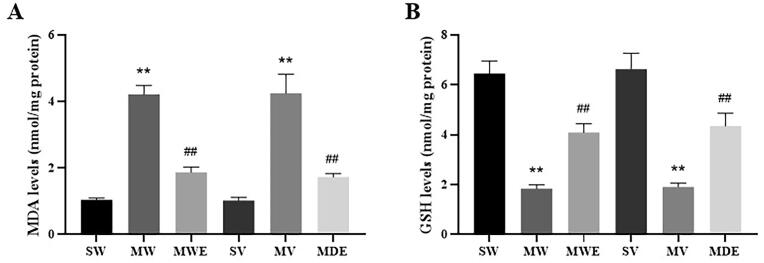
Antioxidant ability of different RPR extracts proved by cerebral cortex data after brain I/R induction. **A** The effect of RPR extract on the level of MDA. **B** The effect of RPR extract on GSH activity. Data are presented as mean ± SD (n = 6). ^**^, *P* < 0.01 compared with the sham group (SW/SV); ^##^, *P* < 0.01 compared with the MCAO group (MW/MV). MDA, malondialdehyde; GSH, reduced glutathione.

Further investigation was carried out to testify whether D-Pro-Sor extract of RPR could induce endogenous antioxidant effects. According to the results in Fig. 6, MCAO induced I/R injuries significantly increased the MDA level and decreased the levels of GSH. Treatment with water and D-Pro-Sor extract of RPR significantly reversed the trends. It was noteworthy that the D-Pro-Sor extract exhibited almost the same effects comparing with aqueous extract of RPR, which suggested that NaDES extracts could potentially be applied ‘directly’ to human or animals without harmful side-effects.

## 4 Conclusion

In conclusion, a highly efficient UAE method for extraction of paeoniflorin derivatives from RPR was established. The selected NaDES extract exhibited strong antioxidant activities *in vitro* and enhanced neuroprotectivity against cerebral I/R injury *in vivo*. Firstly, 27 different NaDESs were prepared and screened as solvent for extraction of PF and GPF. After further optimization of four important factors of UAE, the best NaDES, ChCl-Sor, showed significant advantages over conventional solvents. Under the optimal condition, the yields of PF and GPF were 182.8 mg/g and 77.4 mg/g, respectively. For PF, the results were 112% and 288% better than that of water and methanol; for GPF, the results were 630% and 184% better than that of water and methanol. Four mainstream *in vitro* antioxidant assays were carried out, the results proved the feasibility of NaDESs in active component extraction. *In vivo* experiments revealed that oral administration of RPR extracts can significantly attenuate cerebral I/R induced infarct zone, reduce the content of MDA and increase the GSH concentrations in MCAO rats. The neuroprotectivity of D-Pro-Sor extract exhibited equal or slightly better effect than aqueous extract. The present research proved that NaDESs can not only improve the extraction efficiency, but also as a ‘ready-to-use’ vehicle to exert biological activities of the active ingredients *in vivo*.

## CRediT authorship contribution statement

**Yu Zhao:** Investigation, Writing – original draft. **Haofang Wan:** Methodology, Investigation, Writing – review & editing. **Jiehong Yang:** Methodology. **Yan Huang:** Investigation. **Yu He:** Methodology. **Haitong Wan:** Methodology, Resources, Writing – review & editing, Project administration, Funding acquisition. **Chang Li:** Conceptualization, Investigation, Writing – original draft, Writing – review & editing, Funding acquisition.

## Declaration of Competing Interest

The authors declare that they have no known competing financial interests or personal relationships that could have appeared to influence the work reported in this paper.
