# Supplementary material for: Ultrasound-assisted preparation of ‘Ready-to-use’ extracts from Radix Paeoniae Rubra with natural deep eutectic solvents and neuroprotectivity evaluation of the extracts against cerebral ischemic/ reperfusion injury
Source: Ultrason Sonochem. 2022 Mar 2;84:105968. doi: 10.1016/j.ultsonch.2022.105968 (PMC8908277; doi:10.1016/j.ultsonch.2022.105968)

Preparation of ‘Ready-to-use’ Extracts from Radix Paeoniae Rubra with Natural Deep Eutectic Solvents and Neuroprotectivity Evaluation of the Extracts Against Cerebral Ischemic/ Reperfusion Injury

Yu Zhao, [#1] Haofang Wan, [# 1] Jiehong Yang, [1] Yan Huang, [1]Yu He, [1]Haitong Wan*,[1] Chang Li*,[1]

[1] Zhejiang Chinese Medical University, Hangzhou, 310057, PR China

* Haitong Wan, whtong@126.com; Tel./Fax: +86 571 86613716

* Chang Li, lichang@zju.edu.cn; Tel./Fax: +86 571 86613716

Supporting information

Table of contents **Page**

Table S1. Composition of NaDESs in this work. 2

Table S2. Extraction yields of PF and GPF from RPR using NaDESs and traditional solvents 3

Table S3. Extraction yields of PF and GPF from RPR using ChCl-Sor under different conditions 4

Table S4. Calibration curves and linear ranges for analytes by HPLC 5

Figure S1. HPLC chromatogram of water extract of RPR 6

Figure S2. HPLC chromatogram of ChCl-Sor extract of RPR 6

Figure S3. HPLC chromatogram of methanol extract of RPR 6

Table S1. Composition of NaDESs in this work.

No.	Abbreviation	Type of HBA	Type of HBD	HBA/HBD/(Water)
1	D-Pro-Suc	D-Proline	Sucrose	2:1:10
2	D-Pro-Sor		D-Sorbitol	1:2:4
3	D-Pro-Gly		Glycerol	2:5
4	D-Pro-Ca		Citric Acid	1:1:2
5	D-Pro-Mal		Malonate	1:1:2
6	D-Pro-Mu		1-Methylurea	1:1:2
7	L-Pro-Suc	L-Proline	Sucrose	2:1:10
8	L-Pro-Sor		D-Sorbitol	1:2:4
9	L-Pro-Gly		Glycerol	2:5
10	L-Pro-Ca		Citric Acid	1:1:2
11	L-Pro-Mal		Malonate	1:1:2
12	L-Pro-Mu		1-Methylurea	1:1:2
13	Bet-Glu	Betaine	D-Glucose	1:1:1
14	Bet-Sor		D-Sorbitol	1:1.2:3
15	Bet-Gly		Glycerol	1:2
16	Bet-Ca		Citric Acid	2:1:6
17	Bet-Lac		Lactic Acid	1:1:1
18	Bet-Mu		1-Methylurea	1:1:2
19	ChCl-Glu	Choline Chloride	D-Glucose	1:1:2
20	ChCl-Suc		Sucrose	4:1:3
21	ChCl-Xyl		Xylitol	5:2:5
22	ChCl-Sor		D-Sorbitol	3:1:3
23	ChCl-Gly		Glycerol	1:2
24	ChCl-Ca		Citric Acid	2:1
25	ChCl-Mal		Malonate	1:1
26	ChCl-Mu		1-Methylurea	1:1:1
27	ChCl-Du		N,N’-Dimethylurea	1:1:2

Table S2. Extraction yields of PF and GPF from RPR using NaDESs and traditional solvents

**Entry**	**Solvent**	**PF (mg/g)**	**GPF (mg/g)**
1	water	86.0±9.4	10.6±1.4
2	methanol	47.1±6.3	27.3±3.7
3	D-Pro-Suc	101.0±12.7	52.1±6.0
4	D-Pro-Sor	140.8±4.6	53.6±2.8
5	D-Pro-Gly	102.5±4.8	62.5±9.9
6	D-Pro-Ca	95.4±6.8	38.4±3.2
7	D-Pro-Mal	98.5±33.9	55.1±6.1
8	D-Pro-Mu	103.6±6.3	67.6±0.8
9	L-Pro Suc	51.2±26.3	26.3±10.8
10	L-Pro-Sor	129.1±8.4	49.1±7.7
11	L-Pro-Gly	107.5±5.4	74.4±2.4
12	L-Pro-Ca	110.7±19.7	53.4±3.7
13	L-Pro-Mal	76.4±14.1	56.2±9.8
14	L-Pro-Mu	114.3±16.8	60.9±9.9
15	Bet-Glu	98.8±11.9	48.6±3.8
16	Bet-Sor	96.5±6.6	48.2±4.9
17	Bet-Gly	91.2±9.7	62.9±2.4
18	Bet-Ca	120.5±3.0	52.2±9.1
19	Bet-Lac	74.6±12.3	46.7±1.0
20	Bet-Mu	110.9±20.0	58.2±2.0
21	ChCl-Glu	106.8±15.3	48.3±5.9
22	ChCl-Suc	122.1±8.1	55.9±10.6
23	ChCl-Xyl	114.9±17.4	52.8±14.9
24	ChCl-Sor	133.3±11.4	66.5±2.6
25	ChCl-Gly	90.6±16.5	57.3±0.4
26	ChCl-Ca	132.1±15.6	51.7±3.6
27	ChCl-Mal	97.0±6.2	53.7±6.6
28	ChCl-Mu	113.2±13.3	63.9±5.7
29	ChCl-Du	111.6±5.6	72.0±4.8

Table S3. Extraction yields of PF and GPF from RPR using ChCl-Sor under different conditions (values are expressed as mean±SD)

Extraction Condition		PF (mg/g)	GPF (mg/g)
A. S/L Ratio (mg/mL)	25	133.3±11.4	66.5±2.6
	50	151.6±6.9	69.9±4.6
	100	149.9±13.2	66.2±6.0
	150	148.2±6.0	63.0±3.2
B. DES Content (%)	25	80.6±11.2	30.5±1.8
	50	100.8±14.4	51.3±4.9
	75	133.3±11.4	66.5±2.6
	100	81.7±8.5	50.8±5.6
C. Extraction Time (min)	15	77.4±8.2	47.9±7.7
	30	133.3±11.4	66.5±2.6
	45	115.0±11.4	61.3±6.3
	60	108.4±9.3	59.1±8.1
D. Extraction Temperature(°C)	30	98.5±3.9	52.1±4.8
	40	135.2±10.7	65.3±6.1
	50	133.3±11.4	66.5±2.6
	60	90.1±17.2	50.0±5.6

Table S4. Calibration curves and linear ranges for analytes by HPLC

No.	Wavelength (nm)	Calibration Curve	R^2	Linear Range (μg/mL)
PF	280	Y = 824.14x + 206.75	0.9994	200-1000
GPF		Y = 4664.9x - 28.019	0.9990	31.25-1000

A calibration curve was obtained injecting standard solutions of PF at different concentrations, respectively 1000, 800, 600, 400 and 200 μg/mL. The calibration curve as follow: y = 824.14x+206.75, and the correlation coefficient was 0.9994.

A calibration curve was obtained injecting standard solutions of GPF at different concentrations, respectively 1000, 500, 250, 62.5 and 31.25 μg/mL. The calibration curve as follow: y = 4664.9x-28.019, and the correlation coefficient was 0.9990.

Figure S1. HPLC chromatogram of water extract of RPR (wavelength=280nm)

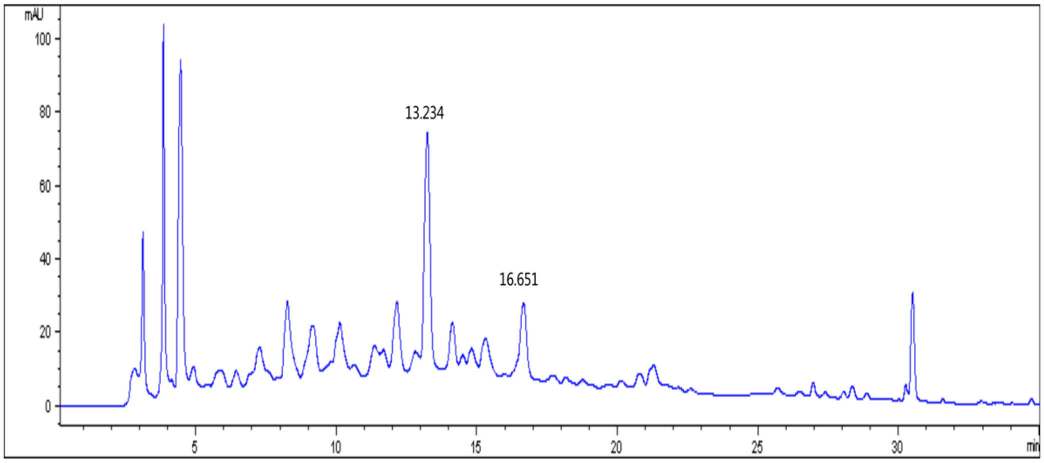

Figure S2. HPLC chromatogram of methanol extract of RPR (wavelength=280nm)

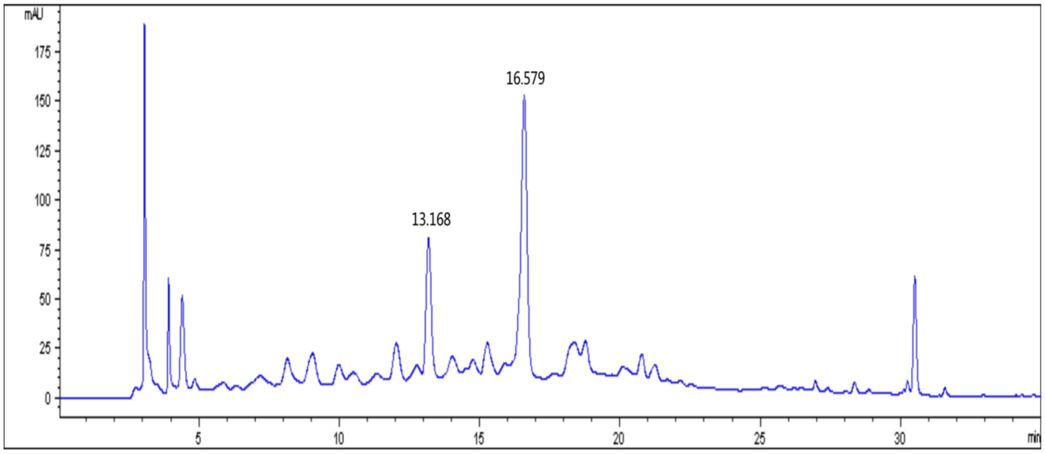

Figure S3. HPLC chromatogram of ChCl-Sor extract of RPR（wavelength=280nm）

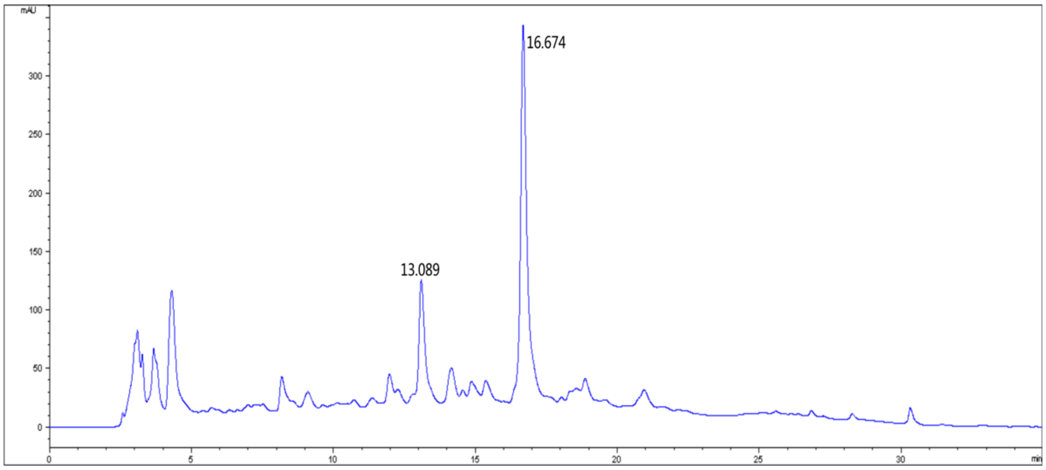

Supplement: Supplementary data 1 [file mmc1.pdf]
